# Effects of age, sex and manual task on hand preference in wild *Rhinopithecus roxellana*

**DOI:** 10.24272/j.issn.2095-8137.2019.023

**Published:** 2018-11-28

**Authors:** Wei-Wei Fu, Xiao-Wei Wang, Cheng-Liang Wang, Hai-Tao Zhao, Yi Ren, Bao-Guo Li

**Affiliations:** 1 Shaanxi Key Laboratory for Animal Conservation, Shaanxi Institute of Zoology, Xi’an Shaanxi 710032, China; 2 Shaanxi Key Laboratory for Animal Conservation, College of Life Sciences, Northwest University, Xi’an Shaanxi 710069, China; 3 Center for Excellence in Animal Evolution and Genetics, Chinese Academy of Sciences, Kunming Yunnan 650223, China

**Keywords:** Handedness, Unimanual reaching, Bimanual coordination, Sex, Age

## Abstract

Golden snub-nosed monkeys (*Rhinopithecus roxellana*), as typical arboreal group-living Old World monkeys, provide an appropriate animal model to research manual laterality and explore the factors affecting hand preference in non-human primates (NHP). This study investigated hand preference based on 63 subjects and four spontaneous manual tasks (including unimanual and bimanual feeding and grooming), and assessed the effects of age, gender and type of task on handedness in *R. roxellana*. A population-level left-handedness was found not only in the bimanual coordinated tasks (bimanual feeding and grooming), but also in one unimanual reaching task (unimanual feeding). There were no significant differences between the sexes in either direction or strength of hand preference among any task. However, a significant difference between adults and juveniles was found in the unimanual feeding task. This is the first report on handedness in unimanual and bimanual feeding tasks that require bipedal posture in wild *R. roxellana.* Furthermore, this study demonstrated spontaneous feeding tasks reported previously only in the quadrupedal posture in this species, supporting the importance of factors such as posture and task complexity in the evolution of primate manual lateralization.

## INTRODUCTION

Hand dominance is defined as a tendency to use one hand over the other to perform most activities. In humans, strong lateralization in handedness was found early and was considered the most obvious example of cerebral lateralization and an exclusive characteristic (Perelle & Ehrman, 2005). Over the past 20 years, however, numerous systematic investigations of handedness in non-human primates (NHP) have been published (Regaiolli et al., 2016). Interestingly, these studies have not shown a similar strong hand preference in NHP as observed in humans, with contradictory findings reported thus far (Regaiolli et al., 2018). Therefore, further study on manual laterality and the factors that affect hand preference in NHP is required.

As typical arboreal group-living Old World primates, golden snub-nosed monkeys (*Rhinopithecus roxellana*, Colobinae, Cercopithecidae) have been studied broadly in regard to hand preference in the wild (Liang & Zhang, 1998; Ma et al., 1988) and in captivity (Zhao et al., 2008, 2010). So far, remarkable achievements, such as the postural origins theory (MacNeilage et al., 1987) and task complexity theory (Fagot & Vauclair, 1991), have been obtained from research on hand preference in non-human primates (NHP). The postural origins theory proposes that arboreal primates preferentially use their left-hand for manual tasks (e.g., grasping food) (MacNeilage et al., 1987). The task complexity theory proposes that preferences and group-level biases of manual laterality more likely appear in bimanual coordinated tasks than in unimanual tasks (Fagot & Vauclair, 1991). Although these two theories are supported in *R. roxellana* (Zhao et al., 2008, 2010), some issues on the handedness of this species remain unclear. First, previous studies on NHP report that bipedal posture can elicit stronger hand preference than quadrupedal posture (Fagot & Vauclair, 1991; MacNeilage, 2008; Sanford et al., 1984). However, unimanual feeding tasks in *R. roxellana* have only been investigated in quadrupedal posture (Zhao et al., 2008), with existing research not yet revealing the influence of bipedal posture on handedness. Second, many studies on NHP species (e.g., chimpanzees (*Pan troglodytes*): Boesch, 1991; orangutans (*Pongo pygmaeus*): Cunningham et al., 1989; Rogers & Kaplan, 1996; *Hylobates* species (*Hylobates syndactylus*, *H. concolor* and *H. lar*): Stafford et al., 1990; tufted capuchin monkeys (*Cebus apella*): Lacreuse & Fragaszy, 1996, Westergaard & Suomi, 1993; Japanese macaques (*Macaca fuscata*): Kubota, 1990 and pig-tailed macaques (*M. leonina*): Zhao et al., 2016) have reported that the strength of hand preference is positively correlated with age. The maturation theory suggests different rates in the maturity of the two hemispheres from the fetal period (Hopkins & Bard, 1993), which has been widely accepted to explain the influence of age on handedness. Based on this theory, different growth rates affect the initial muscle movements of the fetus, with greater strength and unanimous direction of hand preference more likely found in mature individuals (Hopkins & Bard, 1993). Regarding *R. roxellana*, only one captive study has reported no age difference in hand preference (Liang & Zhang, 1998). In addition, although research has reported no significant effect of gender on hand preference in NHP (e.g., Meguerditchian et al., 2015; Meunier & Vauclair, 2007; Wells, 2002), several studies have reported significant differences between the sexes (e.g., Morino et al., 2017; Pan et al., 2011, 2013; Spinozzi & Truppa, 2002). Gender influence on hand preference has only been reported in *R. roxellana* based on one unimanual reaching task (Liang & Zhang, 1998; Ma et al., 1988; Zhao et al., 2008). Thus, the contradictory results of the influence of age and gender on hand preference in *R. roxellana* limit our understanding and highlight the need for further research on handedness in this species under different settings and manual tasks.

In this study, we observed handedness in a population of wild *R. roxellana* in relation to four spontaneous manual tasks (i.e., unimanual feeding, bimanual feeding, unimanual grooming and bimanual grooming). Based on the task complexity theory (Fagot & Vauclair, 1991) and previous reports on bimanual tasks in *R. roxellana* (Zhao et al., 2008, 2010), we hypothesized that hand biases of *R. roxellana* would be similarly evident for bimanual feeding, which has not been reported in this species previously. We also tested whether hand preference for feeding in the bipedal posture would be more biased than that in the quadrupedal posture based on earlier studies (Fagot & Vauclair, 1991; MacNeilage, 2008; Sanford et al., 1984). Importantly, we compared the age and gender differences in hand preference with previous studies on *R. roxellana* (Liang & Zhang, 1998; Ma et al., 1988; Zhao et al., 2008) as well as handedness differences between *R. roxellana* and other NHP, including closely related *R. bieti* (Pan et al., 2011, 2013; Jablonski, 1998). This study on hand preference in *R. roxellana* will provide additional behavioral evidence on the evolution of handedness in NHP.

## MATERIALS AND METHODS

### Study site and species

This study was carried out in Zhouzhi National Nature Reserve on the northern slopes of the Qinling Mountains, Shaanxi, China. Two wild troops of *R. roxellana* live at the study site, i.e., the East Ridge troop and West Ridge troop (WRT). Our focus population was provisioned at Sanchakou (1 646 m a.s.l.) in Gongnigou valley (N33°48′68″, E108°16′18″) within the WRT. Field assistants searched for the focal population and attracted the monkeys to the provisioning site at 0900 h every day. Approximately 200 g of dispersed food (corn, apples and radishes) were provided per monkey per day at three time points (1000 h, 1200 h and 1400 h). Compared with the total daily diet of *R*. *roxellana*, the energy intake of the provisioned food was small and thus its influence on natural behavior was minimal (Li & Zhao, 2007). When the focal population was well habituated to the presence of observers, we identified all focal individuals via physical characteristics and maintained a distance of 5–50 m (Li et al., 2000; Zhang et al., 2006). The focal individuals were divided into four classes: juvenile females (1–3 years), juvenile males (1–5 years), adult females (>5 years) and adult males (>7 years) (Zhang et al., 2006). In total, 63 individuals were involved, consisting of 15 adult males, 27 adult females, 12 juvenile males and 9 juvenile females in 11 one-male multi-female units and the sole all-male unit in the study.

### Data collection

The data were collected over 138 d. A total of 6 030 manual observational data from 63 subjects from September 2010 to May 2011 were collected by focus animal sampling (Altmann, 1974) and behavioral sampling (Martin & Bateson, 2007). Four types of manual task were identified and collected. Under common conditions, one hand is considered dominant and the other hand is considered subordinate. Thus, data were recorded as left-dominant/right-subordinate (L) or right-dominant/left-subordinate (R) in all types of task.

(1) Unimanual feeding: This simple reaching task was observed when a subject fed on the ground in the bipedal posture. The hand that first grasped the food and brought it to its mouth was deemed dominant, whereas the other hand, which was unused or placed on its hind limb, was deemed subordinate. Data were recorded when a focal individual first made contact with food at the same site.

(2) Bimanual feeding: Bimanual feeding was defined as a coordinated bimanual action and was frequently observed when feeding in trees. One hand (subordinate) was used to draw thin branches to the focal individual, while the opposite hand (dominant) was used to pick leaves or bark from the branches and bring them to its mouth (Meguerditchian et al., 2010). If the focal individual continued feeding in the same position, the behavior was only recorded once. If the focal individual changed position, like in the unimanual feeding task, the behavior was recorded a second time.

(3) Unimanual grooming: Grooming is a common behavior in highly social primates. As described previously, unimanual grooming was recorded when the focal individual used only one hand (dominant) to perform grooming, with the other hand (subordinate) placed on its hind limbs or used for postural support (Hopkins et al., 2007). Following the method described by Hopkins et al. (2007), real-time recording over a 5-min observation period was used for data collection on one focal individual. Within the 5-min observation period, data on the focal individual were collected every 15 s. During observation, mouths were sometimes involved in the grooming action, which was ignored due to its low frequency and limited influence on the determination of hand preference. Observation ceased if the focal individual stopped grooming within the 5-min observation period and did not groom in the following 30 s. A new 5-min observation period was continued if the focal individual did not stop grooming after the initial 5-min observation period, and when no other individuals performed grooming within visible distance.

(4) Bimanual grooming: Bimanual grooming was defined as coordinated bimanual action in which the individual used both hands to perform grooming. The dominant hand performed grooming while the subordinate hand was used to hold or stabilize the area around the grooming site (Hopkins et al., 2007). Data collection during bimanual grooming also met the criteria mentioned above for unimanual grooming.

### Data analyses

Three important indexes were used for the determination of hand preference. First, to identify the degree of individual lateral bias, the handedness index (HI) for each focal subject was calculated based on the following formula: (right-hand use–left-hand use)/(right-hand use+left-hand use). The HI varied between −1.0 and 1.0, indicating left- and right-hand bias, respectively. Second, the absolute value of HI (ABS-HI) was used to reflect the strength of individual-level hand preference. Third, the binomial Z-scores were used to determine whether the frequency of left- or right-hand use exceeded that expected by chance (50% right-hand use). Based on the Z-scores, our subjects were categorized as right-handed (z≥1.96), left-handed (z≤−1.96) or ambipreferent (1.96>z>−1.96).

One-sample *t-*tests of the HI scores were used to evaluate whether the group was ambipreferent or lateralized in hand use for each task (Hopkins, 1999) and Mann-Whitney *U*-tests were used for determination of age and gender effects on hand preference. The Spearman correlation test was applied to evaluate the relationship between the number of data points per subject and HI/ABS-HI scores. SPSS v.23.0 and two-tailed significance at *P*≤0.05 were used in all analyses.

## RESULTS

There was no significant correlation between sample size and HI scores (unimanual feeding: *r*=0.327, *P*=0.111; bimanual feeding: *r*=−0.109, *P*=0.699; unimanual grooming: *r*=0.003, *P*=0.992; bimanual grooming: *r*=0.084, *P*=0.598) and ABS-HI scores (unimanual feeding: *r*=−0.324, *P*=0.114; bimanual feeding: *r*=−0.109, *P*=0.699; unimanual grooming: *r*=0.131, *P*=0.655; bimanual grooming: *r*=0.136, *P*=0.409), suggesting that individual differences in the total number of responses did not skew the distribution of handedness values.

For unimanual feeding, the mean number of manual data per subject was 49.92±2.29 (range: 31–67) and the mean HI and ABS-HI scores were −0.21±0.05 (range: −0.51–0.33) and 0.29±0.03 (range: 0.10–0.51), respectively (Table 1); for bimanual feeding, the mean number of manual data per subject was 57.93±6.98 (range: 30–126) and the mean HI and ABS-HI scores were −0.39±0.06 (range: −0.88 to −0.05) and 0.39±0.06 (range: 0.05–0.88), respectively (Table 2); for unimanual grooming, the mean number of manual data per subject was 46.57±5.96 (range: 30–111) and the mean HI and ABS-HI scores were −0.13±0.07 (range: −0.60–0.29) and 0.23±0.05 (range: 0.00–0.60), respectively (Table 3); for bimanual grooming, the mean number of manual data per subject was 77.54±5.66 (range: 30–163) and the mean HI and ABS-HI scores were −0.25±0.04 (range: −0.85–0.54) and 0.33±0.03 (range: 0.01–0.85), respectively (Table 4).

**Table 1:** Hand preference for unimanual feeding task in *R. roxellana* (*n*=25)

Animal ID	Age	Sex	L^a^	R^b^	Percentage (%)^c^	HI	ABS-HI	Z-scores	Preference^d^
BHX	Adult	F	21	12	63.64	−0.27	0.27	−1.57	Ambi
BX	Adult	F	38	23	62.30	−0.25	0.25	−1.92	Ambi
DAH	Adult	F	34	26	56.67	−0.13	0.13	−1.03	Ambi
F2	Adult	F	36	29	55.38	−0.11	0.11	−0.87	Ambi
HUT	Adult	F	36	16	69.23	−0.38	0.38	−2.77	Left
JD	Adult	F	34	26	56.67	−0.13	0.13	−1.03	Ambi
WSF1	Adult	F	27	12	69.23	−0.38	0.38	−2.40	Left
ZFX	Adult	F	20	39	66.10	0.32	0.32	2.47	Right
JB	Adult	M	28	19	59.57	−0.20	0.20	−0.90	Ambi
RX	Adult	M	36	29	55.38	−0.11	0.11	−0.87	Ambi
SH	Adult	M	34	20	62.96	−0.37	0.37	0.32	Ambi
WX	Adult	M	34	28	54.84	−0.10	0.10	−0.64	Ambi
XH	Adult	M	30	20	60.00	−0.19	0.19	−1.66	Ambi
BB	Adult	M	31	16	65.96	−0.32	0.32	−2.19	Left
FP	Adult	M	22	40	64.52	0.29	0.29	2.29	Right
HT	Adult	M	15	30	66.67	0.33	0.33	2.24	Right
JBJ20	Juvenile	F	22	17	56.41	−0.13	0.13	−0.80	Ambi
RXJ20	Juvenile	F	24	13	64.86	−0.30	0.30	−1.81	Ambi
BBJ20	Juvenile	F	26	11	70.27	−0.41	0.41	−2.47	Left
BBJ30	Juvenile	F	27	10	72.97	−0.46	0.46	−2.79	Left
JBJ30	Juvenile	F	30	12	71.43	−0.43	0.43	−2.78	Left
SH	Juvenile	F	49	18	73.13	−0.51	0.51	−3.20	Left
GPJ21	Juvenile	M	22	17	56.41	−0.13	0.13	−0.80	Ambi
JBJ41	Juvenile	M	41	17	70.69	−0.41	0.41	−3.15	Left
RXJ41	Juvenile	M	23	8	74.19	−0.48	0.48	−2.69	Left

^a^: Number of responses made with the left hand. ^b^: Number of responses made with the right hand. ^c^: Percentage of use of the preferred hand. ^d^: Category of hand preference based on Z-scores. M: Male; F: Female.

**Table 2:** Hand preference for bimanual feeding task in *R. roxellana* (*n*=15)

Animal ID	Age	Sex	L^a^	R^b^	Percentage (%)^c^	HI	ABS-HI	Z-scores	Preference^d^
XL	Adult	F	27	21	0.56	−0.13	0.13	−0.87	Ambi
DBC	Adult	F	27	18	0.60	−0.20	0.20	−1.34	Ambi
QQ	Adult	F	24	15	0.62	−0.23	0.23	−1.44	Ambi
RH	Adult	F	21	13	0.62	−0.24	0.24	−1.37	Ambi
XBC	Adult	F	21	19	0.53	−0.05	0.05	−0.32	Ambi
HH	Adult	F	42	24	0.64	−0.27	0.27	−2.22	Left
GTT	Adult	F	42	12	0.78	−0.56	0.56	−4.08	Left
DH	Adult	F	27	3	0.90	−0.80	0.80	−4.38	Left
BB	Adult	M	78	48	0.62	−0.24	0.24	−2.67	Left
JB	Adult	M	78	27	0.74	−0.49	0.49	−4.98	Left
WS	Adult	M	48	27	0.64	−0.28	0.28	−2.42	Left
SH	Adult	M	54	18	0.75	−0.50	0.50	−4.24	Left
WB	Adult	M	45	3	0.94	−0.88	0.88	−6.06	Left
JBJ41	Juvenile	M	33	12	0.73	−0.47	0.47	−3.13	Left
XD	Juvenile	M	33	9	0.79	−0.57	0.57	−3.70	Left

^a^: Number of responses made with the left hand. ^b^: Number of responses made with the right hand. ^c^: Percentage of use of the preferred hand. ^d^: Category of hand preference based on Z-scores. M: Male; F: Female.

**Table 3:** Hand preference for unimanual grooming task in *R. roxellana* (*n*=14)

Animal ID	Age	Sex	L^a^	R^b^	Percentage (%)^c^	HI	ABS-HI	Z-scores	Preference^d^
HB	Adult	F	24	27	0.53	0.06	0.06	0.42	Ambi
HH	Adult	F	18	15	0.55	−0.09	0.09	−0.52	Ambi
WSF1	Adult	F	12	18	0.60	0.20	0.20	1.10	Ambi
WS	Adult	M	23	42	0.65	0.29	0.29	2.36	Right
BB	Adult	M	48	15	0.76	−0.52	0.52	−4.16	Left
JB	Adult	M	33	12	0.73	−0.47	0.47	−3.13	Left
GP	Adult	M	69	42	0.62	−0.24	0.24	−2.56	Left
SH	Adult	M	24	31	0.56	0.13	0.13	0.94	Left
SHJ30	Juvenile	F	21	12	0.64	−0.27	0.27	−1.57	Ambi
RXJ20	Juvenile	F	18	12	0.60	−0.20	0.20	−1.10	Ambi
GPJ20	Juvenile	F	24	6	0.80	−0.60	0.60	−3.29	Left
GPJ21	Juvenile	M	20	16	0.56	−0.11	0.11	−0.67	Ambi
XD	Juvenile	M	21	19	0.53	−0.05	0.05	−0.32	Ambi
WSJ21	Juvenile	M	15	15	0.50	0.00	0.00	0.00	Ambi

^a^: Number of responses made with the left hand. ^b^: Number of responses made with the right hand. ^c^: Percentage of use of the preferred hand. ^d^: Category of hand preference based on Z-scores. M: Male; F: Female.

**Table 4:** Hand preference for bimanual grooming task in *R. roxellana* (*n*=42)

Animal ID	Age	Sex	L^a^	R^b^	Percentage (%)^c^	HI	ABS-HI	Z-scores	Preference^d^
BHX	Adult	F	59	58	0.50	−0.01	0.01	−0.09	Ambi
GPF6	Adult	F	37	31	0.54	−0.09	0.09	−0.73	Ambi
JD	Adult	F	46	55	0.54	0.09	0.09	0.9	Ambi
NZ	Adult	F	32	47	0.59	0.19	0.19	1.69	Ambi
FF	Adult	F	18	51	0.74	0.48	0.48	3.97	Right
KK	Adult	F	41	61	0.60	0.2	0.2	1.98	Right
DBC	Adult	F	78	47	0.62	−0.25	0.25	−2.77	Left
DH	Adult	F	41	16	0.72	−0.44	0.44	−3.31	Left
GGT	Adult	F	23	11	0.68	−0.35	0.35	−2.06	Left
HB	Adult	F	49	23	0.68	−0.36	0.36	−3.06	Left
HH	Adult	F	34	19	0.64	−0.28	0.28	−2.06	Left
HUT	Adult	F	52	32	0.63	−0.24	0.24	−2.09	Left
HX	Adult	F	68	26	0.72	−0.45	0.45	−4.33	Left
LZ	Adult	F	99	38	0.72	−0.45	0.45	−5.21	Left
RH	Adult	F	50	27	0.65	−0.3	0.3	−2.62	Left
WXF1	Adult	F	83	36	0.70	−0.39	0.39	−4.31	Left
WXF2	Adult	F	95	54	0.64	−0.28	0.28	−3.36	Left
YL	Adult	F	67	38	0.64	−0.28	0.28	−2.83	Left
ZFX	Adult	F	92	43	0.68	−0.36	0.36	−4.22	Left
BZT	Adult	M	26	23	0.53	−0.06	0.06	−0.43	Ambi
YB	Adult	M	22	10	0.69	−0.38	0.38	−2.12	Ambi
FP	Adult	M	16	54	0.77	0.54	0.54	4.54	Right
JB	Adult	M	29	49	0.63	0.26	0.26	2.26	Right
FQ	Adult	M	29	15	0.67	−0.33	0.33	−1.98	Left
GP	Adult	M	47	16	0.75	−0.49	0.49	−3.91	Left
SH	Adult	M	107	56	0.66	−0.31	0.31	−3.99	Left
WS	Adult	M	34	15	0.69	−0.39	0.39	−2.71	Left
XH	Adult	M	35	18	0.66	−0.32	0.32	−2.34	Left
BBJ40	Juvenile	F	32	22	0.59	−0.19	0.19	−1.36	Ambi
SHJ40	Juvenile	F	28	13	0.68	−0.37	0.37	−2.34	Left
AMUJ412	Juvenile	M	32	23	0.58	−0.16	0.16	−1.21	Ambi
BBJ20	Juvenile	M	19	21	0.53	0.05	0.05	0.32	Ambi
GPJ31	Juvenile	M	20	10	0.67	−0.33	0.33	−1.83	Ambi
GPJ20	Juvenile	M	38	3	0.93	−0.85	0.85	−5.47	Left
HSJ20	Juvenile	M	75	29	0.72	−0.44	0.44	−4.51	Left
JBJ41	Juvenile	M	83	54	0.61	−0.21	0.21	−2.48	Left
RXJ41	Juvenile	M	57	21	0.73	−0.46	0.46	−4.08	Left
RXJ410	Juvenile	M	25	11	0.69	−0.39	0.39	−2.33	Left
SHJ20	Juvenile	M	26	6	0.81	−0.63	0.63	−3.54	Left
WSSJ	Juvenile	M	33	17	0.66	−0.32	0.32	−2.26	Left

^a^: Number of responses made with the left hand. ^b^: Number of responses made with the right hand. ^c^: Percentage of use of the preferred hand. ^d^: Category of hand preference based on Z-scores. M: Male; F: Female.

**Figure 1:**
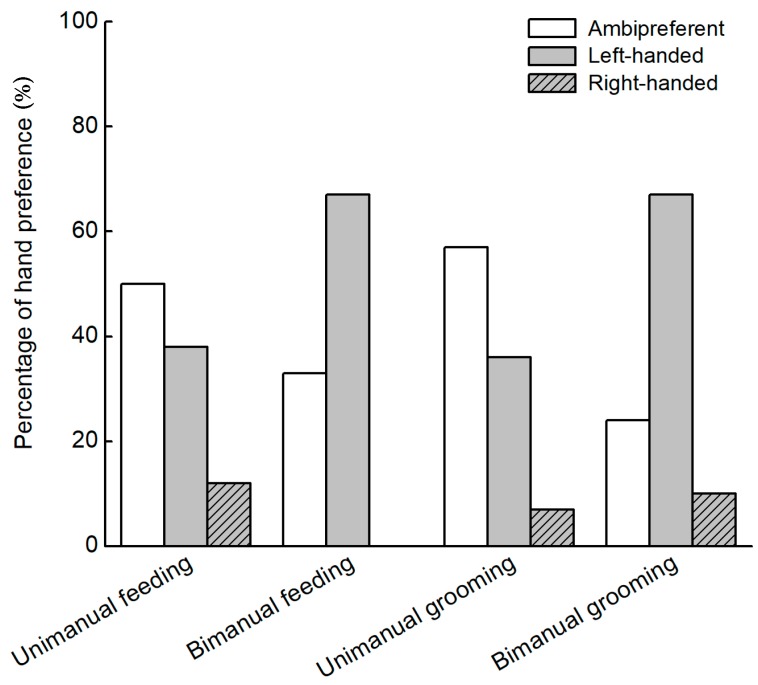
Percentage of subjects exhibiting right-hand, left-hand and ambidextrous preference in unimanual feeding (*n*=25), bimanual feeding (*n*=15), unimanual grooming (*n*=14) and bimanual grooming (*n*=42) tasks in *R. roxellana*

When using Z-scores as the index for hand preference classification to determine individual-level hand preference, 38% individuals in unimanual feeding, 67% in bimanual feeding, 36% in unimanual grooming and 67% in bimanual grooming showed left-handed preference (Figure 1). No individual showed right-handed preference in bimanual feeding, and only 12%, 7% and 10% showed right-handed preference in unimanual feeding, unimanual grooming and bimanual grooming, respectively. One sample *t*-tests on the HI scores revealed significant population-level left-hand preferences for unimanual feeding task (*t*_24_=−4.447, *P*<0.001), bimanual feeding task (*t*_14_=−6.325, *P*<0.001) and bimanual grooming task (*t*_41_=−5.722, *P*<0.001) but not for unimanual grooming task (*t*_13_=−1.859; *P*=0.086).

**Figure 2:**
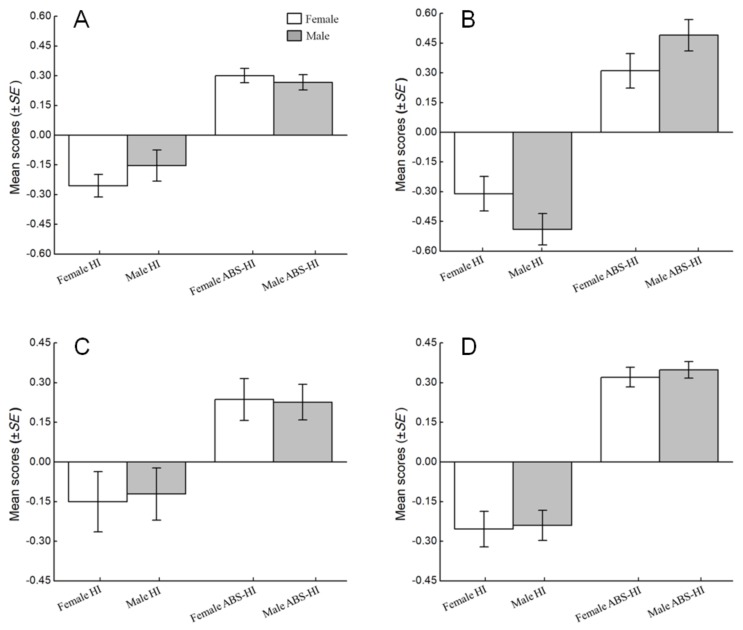
Influence of sex on hand preference among four types of task in *R. roxellana*

The effects of sex on the direction and strength of hand preference were assessed using the HI and ABS-HI scores, respectively. For unimanual feeding, the mean HI score per subject was −0.26, *SE*=0.06 for females, −0.15, *SE*=0.08 for males; the mean ABS-HI score per subject was 0.30, *SE*=0.04 for females and 0.27, *SE*=0.04 for males; there were no differences between the sexes in HI (*N_a_*=14, *N_b_*=11; *U*=56.5, *P*=0.261) or ABS-HI scores (*N_a_*=14, *N_b_*=11; *U*=64.00, *P*=0.475) (Figure 2A). For bimanual feeding, the mean HI score per subject was −0.31, *SE*=0.09 for females and −0.49, *SE*=0.08 for males; the mean ABS-HI score per subject was 0.31, *SE*=0.09 for females and 0.49, *SE*=0.08 for males; no differences were found between the sexes in HI (*N_a_*=8, *N_b_*=7; *U*=12.50, *P*=0.073) or ABS-HI scores (*N_a_*=8, *N_b_*=7; *U*=12.50, *P*=0.073) (Figure 2B). For unimanual grooming, the mean HI score per subject was −0.15, *SE*=0.11 for females and −0.12, *SE*=0.09 for males; the mean ABS-HI score per subject was 0.24, *SE*=0.08 for females and 0.23, *SE*=0.07 for males; no differences were found between the sexes in HI (*N_a_*=6, *N_b_*=8; *U*=22.00, *P*=0.795) or ABS-HI scores (*N_a_*=6, *N_b_*=8; *U*=24.00, *P*=1.000) (Figure 2C). For bimanual grooming, the mean HI score per subject was −0.24, *SE*=0.05 for females and −0.25, *SE*=0.07 for males; the mean ABS-HI score per subject was 0.32, *SE*=0.04 for females and 0.35, *SE*=0.03 for males; there were no differences between the sexes in HI (*N_a_*=27, *N_b_*=17; *U*=186.00, *P*=0.497) or ABS-HI scores (*N_a_*=25, *N_b_*=17; *U*=173.00, *P*=0.311) (Figure 2D).

A: Unimanual feeding task; B: Bimanual feeding task; C: Unimanual grooming task; D: Bimanual grooming task. Values are means±*SE*.

**Figure 3:**
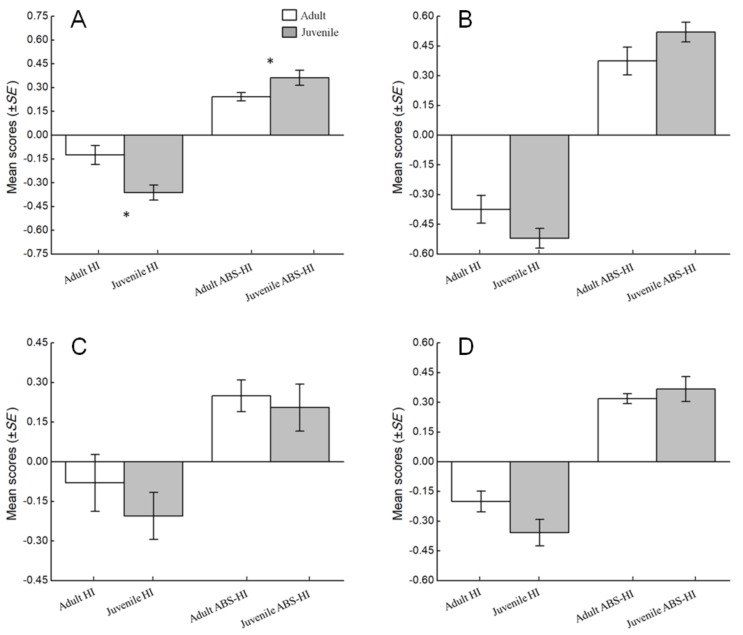
Influence of age on hand preference among four types of task in *R. roxellana*

The effects of age on hand preference were also assessed using the HI and ABS-HI scores. For unimanual feeding, the mean HI score per subject was −0.16, *SE*=0.06 for adults and −0.36, *SE*=0.05 for juveniles, and juveniles showed more left-handedness than adults (*N_a_*=30, *N_b_*=12; *U*=137.50, *P*=0.236); the mean ABS-HI score per subject was 0.24, *SE*=0.03 for adults and 0.36, *SE*=0.05 for juvenile, and juveniles showed a stronger hand preference than adults (*N_a_*=16, *N_b_*=9; *U*=30.00, *P*=0.017) (Figure 3A). For bimanual feeding, the mean HI score per subject was −0.37, *SE*=0.07 for adults and −0.52, *SE*=0.05 for juveniles; the mean ABS-HI score per subject was 0.37, *SE*=0.07 for adults and 0.52, *SE*=0.05 for juveniles; there were no differences between juveniles and adults in HI (*N_a_*=13, *N_b_*=2; *U*=7.00, *P*=0.308) or ABS-HI scores (*N_a_*=13, *N_b_*=2; *U*=7.00, *P*=0.308) (Figure 3B). For unimanual grooming, the mean HI score per subject was −0.08, *SE*=0.10 for adults and −0.21, *SE*=0.08 for juveniles; the mean ABS-HI score per subject was 0.25, *SE*=0.06 for adults and 0.20, *SE*=0.08 for juveniles; no differences were found between juveniles and adults in HI (*N_a_*=8, *N_b_*=6; *U*=16.00, *P*=0.300) or ABS-HI scores (*N_a_*=8, *N_b_*=6; *U*=19.50, *P*=0.559) (Figure 3C). For bimanual grooming, the mean HI score per subject was −0.20, *SE*=0.05 for adults and −0.36, *SE*=0.07 for juveniles; the mean ABS-HI score per subject was 0.32, *SE*=0.03 for adults and 0.37, *SE*=0.06 for juveniles; there were no differences between juveniles and adults in HI (*N_a_*=30, *N_b_*=12; *U*=137.50, *P*=0.236) or ABS-HI scores (*N_a_*=30, *N_b_*=12; *U*=167.00, *P*=0.717) (Figure 3D).

A: Unimanual feeding task; B: Bimanual feeding task; C: Unimanual grooming task; D: Bimanual grooming task. Values are means±*SE*. * indicates significant difference between adults and juveniles (*P*<0.05).

## DISCUSSION

Bimanual feeding was the first task reported in hand preference research and the third spontaneous bimanual task, after bimanual mount reaching and grooming, reported in *R. roxellana* (Zhao et al., 2008, 2010). Population-level hand preference in bimanual feeding and grooming tasks supports our hypothesis that hand preference in bimanual feeding would be similar to that in bimanual grooming (Zhao et al., 2010). In addition, the population-level hand preference in bimanual feeding and bimanual grooming, but not in unimanual grooming, are in accordance with the task complexity theory (Fagot & Vauclair, 1991). Based on that theory, population-level hand preference is not elicited in simple reaching tasks (e.g., unimanual grooming), which has also been reported in other studies (Meguerditchian et al., 2010; Zhao et al., 2008, 2010). Therefore, our unimanual feeding results are not consistent with the task complexity theory. This could be explained by the relative complexity of our unimanual feeding task, in which the main provisioned food (corn) was very small and light, requiring precision grasping. Accurate operation is a determinant for complex tasks (Blois-Heulin et al., 2006; Meunier & Vauclair, 2007). Therefore, feeding on corn might be motorically more complex than feeding on other foods (e.g., apples and radishes), and is thus more likely to elicit greater hand preference.

In the current study, *R. roxellana* exhibited more left-handedness than right-handedness in all four tasks, which supports the postural origins theory (MacNeilage et al., 1987). Moreover, the finding that the proportion of ambidextrously-handed individuals was higher than left-handed or right-handed individuals in both unimanual tasks (based on individual Z-scores) may be linked to the increase in time spent on the ground. This is because increased activities on the ground can relax selective pressure on the strong hand to support body weight and increase opportunities to use both hands (Morino et al., 2017).

We also reported unimanual feeding in the bipedal posture and a population-level hand preference in this posture, different from individual-level hand preference observed in the quadrupedal posture for the same species (Zhao et al., 2008). This finding supports our hypothesis and is in accordance with other studies reporting that bipedal posture can elicit stronger hand preference than quadrupedal posture (Fagot & Vauclair, 1991; MacNeilage, 2008; Sanford et al., 1984). The reason for the increase of hand preference in the bipedal posture is that this posture can constrain the choice of left or right hand due to postural demands, which results in the hand being used to support the body not being used for reaching (Fagot & Vauclair, 1991; MacNeilage, 2008; Sanford et al., 1984). However, previous research on *R. bieti* implies increased right-hand bias in the bipedal posture compared to that in the quadrupedal posture (Pan et al., 2013), in disagreement with our result. These discrepancies may be related to the different species or different wild and captive settings.

There were no differences in the direction or strength of hand preference between the sexes in any task in *R. roxellana*, which agrees with previous research on the same species (in captivity: Liang & Zhang, 1998; in the wild: Zhao et al., 2008) and is also supported by several other studies (e.g., chimpanzees: Chapelain & Hogervorst, 2009; Corp & Byrne, 2004; gorillas (*Gorilla gorilla*): Meguerditchian et al., 2010; white-faced capuchins (*C. capucinus*): Meunier & Vauclair, 2007; pig-tailed macaques (*M. leonina*): Zhao et al., 2016). These findings reveal similar hemisphere specialization between the sexes in NHP. However, previous findings on *R. bieti* suggest that males prefer the left hand significantly more often than do females, with the sex effect on hand preference even shown to be task- (Pan et al., 2011) and posture-specific (Pan et al., 2013). The differential findings between *R. roxellana* and *R. bieti* might correlate to the sexual dimorphism between the two species (Pan et al., 2011). As *R. bieti* is sexually more dimorphic than *R. roxellana* (Jablonski & Pan, 1995), this might lead to a stronger sex effect on hand preference (Pan et al., 2011).

The current study also showed no significant differences between juvenile and adult *R. roxellana* in the direction or strength of hand preference for all tasks, except for unimanual feeding, in which more left-handedness and greater handedness were found in juveniles than in adults. Based on Morino et al. (2017), an increase in time spend on the ground by individuals can increase opportunities to use both hands (Morino et al., 2017). Therefore, the increase in time spent on the ground by adults resulted in greater liberation of their right hand compared with that of juveniles, which may explain the more ambidextrous adults (62.5%) than juveniles (33.3%) (Table 1) in this arboreal primate. Correspondingly, the greater ambidexterity in adults led to a decrease in the rate of one hand usage and showed a weakening in the strength of hand preference compared with that observed in juveniles. Moreover, our findings on the effects of age on hand preference did not support the maturation theory (Hopkins & Bard, 1993) and the difference may relate to the selection of subjects. Unfortunately, we failed to collect data on infant behaviors in the study. The main reason is that infants rarely groomed others or themselves and often fed on the ground alone. Thus, the existing data did not allow us to further analyze age influence on hand preference and prevented further explanation of the ontogenetic development of hand preference in NHP (Hopkins & Bard, 1993). A manual task shared among all age-classes will be needed in future studies of this arboreal primate species.

In conclusion, this is the first report on spontaneous unimanual and manual feeding in the bipedal posture in wild *R. roxellana*, with results showing population-level handedness in both tasks. The unimanual feeding results may be linked to the need for accurate operation when feeding on corn on the ground and the bipedal posture therefore elicited greater hand preference than quadrupedal posture. A population-level handedness was also found in manual grooming, but not in unimanual grooming, which is in accordance with previous study on *R. roxellana* (Zhao et al., 2010), with both supporting the theory of task complexity (Fagot & Vauclair, 1991). Moreover, there were no significant differences in the direction or strength of hand preference between the sexes in any of the tasks. Furthermore, a significant effect of age on the direction and strength of hand preference was only found in unimanual feeding. We did not compare the level of individual hand specialization in different manual tasks because only three out of 63 individuals (5%) were observed in all four tasks. Further study should focus on individual hand specialization among tasks and investigate tasks shared among all age-classes in *R. roxellana* to clarify hand preference in NHP and facilitate comparative research between non-human and human primates.
